# High titers of thyroid peroxidase antibodies as a potential risk factor for osteoporosis: A cross-sectional NHANES study and bidirectional Mendelian randomization analysis

**DOI:** 10.1097/MD.0000000000049917

**Published:** 2026-07-24

**Authors:** Rujie Jiang, Shuyu Yu, Jie You, Xiaowen Zhong, Juntao Liu, Chuan Wan, Xiaochao Xie, Yanan Sun, Jiapeng Niu, Fang Wang

**Affiliations:** a Department of Endocrinology and Metabolism, The Affiliated Hospital of Qingdao University, Qingdao, People’s Republic of China; b Department of Endocrinology, Qingdao Hiser Hospital Affiliated of Qingdao University (Qingdao Traditional Chinese Medicine Hospital), Qingdao, People’s Republic of China.

**Keywords:** antibody titers, bone mineral density, fracture risk, Hashimoto’s thyroiditis, osteoporosis, thyroglobulin antibody, thyroid peroxidase antibody

## Abstract

This study investigates the associations between thyroid autoimmunity in Hashimoto’s thyroiditis (HT), particularly thyroid peroxidase antibody (TPOAB) and thyroglobulin antibody (TGAB) titers, and bone mineral density (BMD) and fracture risk, and explores potential causal relationships using Mendelian randomization (MR) analysis. Data were obtained from nationally representative adults in the NHANES 2007–2010 cohort. Associations between thyroid autoantibody levels, BMD at the femoral neck and lumbar spine, and fracture risk were analyzed using multivariable linear and logistic regression with adjustments for demographic, lifestyle, and biochemical factors. A 2-sample bidirectional MR analysis was performed to assess the causal link between HT and osteoporosis (OP). After accounting for confounders, positive status and higher titers of TPOAB were significantly linked to decreased BMD at the femoral neck and lumbar spine (*P* < .05). TGAB positivity was independently associated with lower lumbar spine BMD, while the association for femoral neck BMD disappeared after multivariable adjustment. TPOAB seropositivity was associated with elevated spinal fracture risk in a dose–dependent manner, and receiver operating characteristic (ROC) analysis showed a moderate discriminatory ability of TPOAB titers for spinal fracture. MR analyses indicated a potential causal association between HT and OP, and sensitivity analyses showed generally consistent directions of effect and did not indicate substantial heterogeneity or horizontal pleiotropy. Increased thyroid autoantibodies, notably higher TPOAB titers, correlate with bone loss and elevated spinal fracture risk, and relevant genetic data suggest a causal association. Thyroid autoimmunity, particularly raised TPOAB levels, may aid the identification of people with low BMD. Additional prospective research is required to determine whether antibody titers enhance fracture risk stratification and act as modifiable therapeutic targets.

## 1. Introduction

Osteoporosis (OP) is a prevalent metabolic bone disorder characterized by decreased bone mass and microarchitectural deterioration, leading to increased fragility and fracture risk.^[1]^ It imposes substantial physical and psychosocial burdens and represents a major public health concern.^[2]^ Three decades ago, the World Health Organization introduced BMD standard deviation (SD)-based scoring as the diagnostic framework for OP, which has since become a cornerstone of clinical assessment. The discovery of receptor activator of nuclear factor-κB ligand (RANKL) as a key osteoclastogenic factor expressed on T cells shifted attention to the role of immune activation in bone loss.^[3]^ Excessive immune responses characteristic of autoimmune diseases have been shown to accelerate bone resorption and impair bone formation.^[4,5]^ As research into osteogenesis, bone remodeling, and inflammatory immunity has deepened, the discipline investigating the interface of the immune system and bone – termed “osteoimmunology” – emerged, as proposed by Arron and Cho.^[6]^

HT, a T-cell-mediated autoimmune thyroid disorder marked by lymphocytic infiltration and production of TPOAB and TGAB,^[7,8]^ is one of the most prevalent autoimmune diseases. Although HT is traditionally classified as an organ-specific autoimmune endocrine disease, accumulating evidence indicates that thyroid autoimmunity is frequently accompanied by systemic low-grade inflammation and may lead to a variety of extrathyroidal manifestations.^[9,10]^ High-titer autoantibodies can activate the complement system and recruit immune cells, which further aggravate pro-inflammatory activation of neutrophils. This self-amplifying inflammatory loop may serve as a key pathway linking HT-related immune dysregulation to aberrant bone metabolism.^[11]^ Immune cells originate from the bone marrow and share the same developmental microenvironment with osteoblasts and osteoclast precursors, providing a biological basis for the crosstalk between the immune system and bone tissue.^[12]^ Although thyroid hormones are essential for skeletal homeostasis, accumulating evidence suggests that autoimmune mechanisms in HT may independently affect bone metabolism through chronic inflammation and cytokine activation, such as interferon-γ, tumor necrosis factor-α (TNF-α), and interleukin-6 (IL-6).^[13]^ Prior research has reported an increased risk of osteoporosis among postmenopausal women with HT and identified TPOAB positivity as a risk factor for fracture,^[14]^ but small sample sizes and confounding have limited causal inference.

The National Health and Nutrition Examination Survey (NHANES) provides large, nationally representative data that allow robust evaluation of associations between thyroid autoimmunity and bone health. In addition, MR, which uses genetic variants as instrumental variables, offers a complementary approach to assess potential causal relationships with reduced bias from confounding or reverse causation. By integrating NHANES-based observational analyses with genetic instrumental variable analysis, this study aimed to bridge the inferential gap between antibody-level associations and disease-level causal evidence.

In this study, our observational analysis assessed whether measured TPOAB and TGAB titers were associated with BMD and fracture risk, while excluding confounding by thyroid-stimulating hormone (TSH) and free thyroxine (FT4). In parallel, we conducted a 2-sample MR analysis examining whether genetic liability to HT was associated with osteoporosis risk.

## 2. Materials and methods

### 2.1. Ethics statement

This study utilized data from NHANES, which adheres to strict ethical standards. Informed consent was obtained from all participants, ensuring the protection and anonymization of their personal information. The MR analysis relied on publicly available summary statistics from GWAS, which were ethically approved during data collection. Thus, the study complies with all ethical guidelines, respecting participants’ rights and privacy.

### 2.2. Study population in NHANES

NHANES employs a stratified, multistage, probability cluster sampling design to obtain a nationally representative sample of the non-institutionalized US civilian population. Demographic, socioeconomic, dietary, and health-related information is collected via standardized questionnaires. Physical examinations and laboratory assessments are conducted in the Mobile Examination Center (MEC). Because BMD data were not available for the 2011–2012 cycle, our inclusion criteria comprised participants enrolled at the NHANES MEC during 2007 to 2010. Exclusion criteria were: missing data on femoral neck or lumbar spine BMD and/or thyroid-related variables; abnormal thyroid function or a history of thyroid cancer, hyperthyroidism, or hypothyroidism; and previously used thyroid medication or glucocorticoids or age < 20 years. Ultimately, 3094 participants with complete BMD and thyroid data were included in the analysis. These inclusion and exclusion criteria were designed to minimize confounding effects from overt thyroid dysfunction and glucocorticoid exposure, both of which can independently alter bone metabolism. Nevertheless, this selection restricts the generalizability of our findings to the overall population of patients with HT. The study protocol and procedures were approved by the National Center for Health Statistics (NCHS) Research Ethics Review Board, and written informed consent was obtained from all participants. NHANES operates in 2-year cycles; details are available at: https://wwwn.cdc.gov/Nchs/Nhanes/.

### 2.3. Measurement of thyroid variables, BMD, and fracture in NHANES

Thyroid measures were obtained using standardized laboratory methods. TGAB and TPOAB were measured with a sequential, 2-step immunoenzymatic “sandwich” assay; TSH was measured using a third-generation, 2-site immunoenzymatic “sandwich” assay; and FT4 was determined by a 2-step enzyme immunoassay.^[15]^ Reference ranges were: TGAB, 0 to 4 IU/mL; TPOAB, 0 to 9 IU/mL; TSH, 0.34 to 5.6 mIU/mL; and FT4, 0.6 to 1.6 ng/dL. Accordingly, we classified antibody status as follows: TGAB (+) (TGAB > 4 IU/mL) vs. TGAB (−) (TGAB ≤ 4 IU/mL); TPOAB (+) (TPOAB > 9 IU/mL) vs. TPOAB (−) (TPOAB ≤ 9 IU/mL).

BMD was assessed in the NHANES MEC using dual-energy x-ray absorptiometry (DXA) scanning.^[16]^ We extracted complete data for femoral neck and lumbar spine BMD, expressed in g/cm^2^. History of fracture was based on a self-report questionnaire, which was recorded in the Questionnaire Data. Participants were asked whether their doctors ever told them that they had fractured their hip, spine, or wrist.

### 2.4. Other covariates in NHANES

To control for potential confounding, we adjusted for a comprehensive set of covariates, including age, gender, race, poverty status, educational attainment, body mass index (BMI), alcohol consumption, smoking status, physical activity, hemoglobin, vitamin D intake, alkaline phosphatase, serum phosphorus, serum calcium, C-reactive protein (CRP), and the presence of diabetes and kidney disease. Poverty-to-income ratio (PIR) was calculated as total family (or individual) income divided by the poverty threshold specific to the survey year. Alcohol consumption categories were defined as follows: Heavy drinking: men > 30 g/d; women > 20 g/d. Moderate drinking: men ≥ 20 to < 30 g/d; women ≥ 10 to < 20 g/d. Light drinking: men < 20 g/d; women < 10 g/d. Never drinking: 0 g/d. Smoking status: Former smoker: >100 cigarettes in lifetime and currently not smoking. Never smoker: <100 cigarettes in lifetime. Current smoker: >100 cigarettes in lifetime and currently smoking. Physical activity was self-reported using the Global Physical Activity Questionnaire (GPAQ), which assesses time spent in vigorous- and moderate-intensity activities across work, transportation, and leisure in a typical week. Using NHANES-recommended conversion factors, we computed metabolic equivalent (MET)-minutes and categorized PA as < 600, 600–7999, and ≥ 8000 MET-min/wk.^[17]^

### 2.5. Mendelian randomization analysis

In the 2-sample MR study, we used single-nucleotide polymorphisms (SNPs) as instrumental variables (IVs) under 3 core assumptions^[18,19]^: Relevance: the IVs must be robustly associated with the exposure (HT). Independence: the IVs are independent of confounders of the HT–OP relationship. Exclusion restriction: the IVs influence the outcome (OP) only through the exposure (HT), that is, they have no direct association with OP other than via HT. Genetic variants associated with HT were derived from the meta-analysis of GWASs by Sakaue et al,^[20]^ comprising 3,95,640 individuals of European ancestry (15,653 cases and 3,79,986 controls). Summary statistics for OP were obtained from the FinnGen GWAS, including 3203 cases and 2,09,575 controls. All contributing studies received approval from local institutional review boards, and all participants provided informed consent; no additional ethical approval was required for the present secondary analyses.

To identify valid genetic instruments for estimating the causal effect of the exposure (HT) on the outcome (OP), we: applied a genome-wide significance threshold of *P* < 5 × 10^−8^ to select SNPs strongly associated with HT; performed linkage disequilibrium (LD) clumping to ensure independence among instruments using *r*^2^ < 0.001 and a 10,000 kb window; and queried each candidate SNP in the PhenoScanner database to exclude variants strongly associated with potential confounders or with OP itself. Following these steps, 11 SNPs were retained for subsequent causal inference analyses. We further performed reverse MR analyses, with OP set as the exposure and HT as the outcome, to test for potential reverse causality.

### 2.6. Statistical analysis

For baseline characteristics, categorical variables are presented as survey-weighted percentages, and continuous variables as survey-weighted means (standard errors, SE). For the NHANES analyses, we fitted survey-weighted, multivariable regression models to assess the associations of thyroid autoantibodies (TPOAB, TGAB) with femoral neck and lumbar spine BMD, as well as the risks of wrist, spinal, and hip fractures. Three covariate-adjustment models were evaluated: Model 1: unadjusted. Model 2: adjusted for age, gender, race, PIR, BMI, alcohol consumption, smoking status, and physical activity. Model 3: additionally adjusted (beyond model 2) for hemoglobin, dietary vitamin D intake, ALP, serum phosphorus, serum calcium, TSH, FT4, and CKD status. We also performed stratified analyses according to antibody titers. Seropositive participants were further divided into low-titer and high-titer groups using a cutoff value of 600 IU/mL. This threshold was predefined at the study design stage, referring to the upper reporting limit for markedly elevated thyroid autoantibodies in our institutional laboratory. Given the lack of universally accepted thyroid antibody cutoffs for stratifying skeletal risk, this grouping approach was adopted merely as an exploratory standard for clinical interpretation. Results are reported as β coefficients, odds ratios (ORs), and their 95% confidence intervals (CIs). To further explore potential heterogeneity in the effects of the 2 thyroid autoantibodies on BMD, we performed subgroup analyses stratified by age, gender, race, PIR, educational attainment, BMI, alcohol consumption, smoking status, and physical activity. ROC curve analysis was used to assess the discriminatory value of TPOAB titers for spinal fracture. Given the complex, multistage probability sampling design of NHANES, survey weights were applied in all analyses: wtmec2yr for 2007 to 2008 and wtsa2yr for 2009 to 2010.

In the 2-sample MR analysis, we used inverse-variance weighting (IVW) as the primary method to estimate the causal association between genetically predicted HT and OP risk. IVW meta-analyzes SNP-specific Wald ratios (computed as the SNP-outcome β divided by the SNP-exposure β) and fits a weighted regression of SNP-outcome effects on SNP-exposure effects with the intercept constrained to 0; the resulting slope provides the overall causal estimate.^[19]^ To evaluate the robustness of IVW findings, we conducted 4 complementary MR methods: MR-Egger, weighted median, weighted mode, and simple mode. We assessed heterogeneity and horizontal pleiotropy using Cochran *Q* and MR-PRESSO, respectively, and performed leave-one-out analyses to determine whether results were driven by any single variant.

All statistical analyses were conducted in R (version 4.5.0; R Foundation for Statistical Computing). *P* < .05 was considered statistically significant.

## 3. Results

### 3.1. Characteristics of the study population

Table S1, Supplemental Digital Content 1 presents the clinical and laboratory characteristics of participants. Femoral neck and lumbar spine BMD were divided into tertiles (Q1, Q2, Q3). Overall, individuals in the low BMD group (Q1) were older, predominantly female, and had a higher proportion of non-Hispanic Whites. They also had lower BMI, lower participation in physical activity, lower vitamin D intake, a higher prevalence of CKD, and elevated ALP levels. Compared with the high BMD group, the low BMD group had a greater proportion of TGAB- and TPOAB-positive individuals and higher TPOAB antibody titers, whereas TGAB titers did not differ significantly between groups.

### 3.2. Associations of TPOAB and TGAB with BMD in NHANES

Tables 1 and 2 summarize the associations of thyroid autoantibody positivity and titers with BMD at the femoral neck and lumbar spine. In the unadjusted analyses, participants positive for either TPOAB or TGAB had significantly lower BMD than seronegative individuals (*P* < .05). After adjustment for demographic variables, socioeconomic status, lifestyle factors, and biochemical parameters, the association between TGAB positivity and femoral neck BMD lost statistical significance (*P* = .410). In contrast, TGAB remained significantly associated with lower lumbar spine BMD (model 3: β = −0.023 [95% CI: −0.066 to −0.016], *P* = .003); TPOAB was significantly associated with reduced femoral neck BMD (model 3: β = −0.031 [95% CI: −0.047 to −0.014], *P* = .001) and lumbar spine BMD (model 3: β = −0.035 [95% CI: −0.053 to −0.017], *P* < .001). Notably, individuals who tested positive for both TPOAB and TGAB exhibited the lowest BMD values in all models, including femoral neck BMD (model 3: β = −0.037 [95% CI: −0.060 to −0.015], *P* = .003) and lumbar spine BMD (model 3: β = −0.052 [95% CI: −0.080 to −0.025], *P* < .001).

**Table 1:** Association between TPOAB and TGAB seropositivity with BMD.

Femoral Neck BMD	Model 1: β (95% CI)	*P*	Model 2: β (95% CI)	*P*	Model 3: β (95% CI)	*P*
TGAB (−)	Reference		Reference		Reference	
TGAB (+)	−0.049 (−0.077, −0.021)	**.001**	−0.023 (−0.045, 0.010)	.260	−0.022 (−0.043, 0.020)	.410
TPOAB (−)	Reference		Reference		Reference	
TPOAB (+)	−0.066 (−0.082, −0.050)	**<.001**	−0.032 (−0.049, −0.015)	**<.001**	−0.031 (−0.047, −0.014)	**.001**
TGAB&TPOAB (−)	Reference		Reference		Reference	
TGAB&TPOAB (+)	−0.058 (−0.088, −0.027)	**<.001**	−0.040 (−0.064, −0.016)	**.002**	−0.037 (−0.060, −0.015)	**.003**
Lumbar spine BMD	Model 1: β (95% CI)	*P*	Model 2: β (95% CI)	*P*	Model 3: β (95% CI)	*P*
TGAB (−)	Reference		Reference		Reference	
TGAB (+)	−0.05 (−0.082, −0.027)	**<.001**	−0.039 (−0.064, −0.014)	**.003**	−0.023 (−0.066, −0.016)	**.003**
TPOAB (−)	Reference		Reference		Reference	
TPOAB (+)	−0.057 (−0.074, −0.040)	**<.001**	−0.047 (−0.062, −0.032)	**<.001**	−0.035 (−0.053, −0.017)	**<.001**
TGAB & TPOAB (−)	Reference		Reference		Reference	
TGAB & TPOAB (+)	−0.064 (−0.093, −0.034)	**<.001**	−0.050 (−0.077, −0.023)	**<.001**	−0.052 (−0.080, −0.025)	**<.001**

Values are β coefficients and 95% CIs for BMD. Antibody-negative participants were used as the reference group. Model 1 was unadjusted. Model 2 was adjusted for age, gender, race, PIR, BMI, alcohol consumption, smoking status, and physical activity. Model 3 was additionally adjusted for Hb, dietary vitamin D intake, ALP, serum phosphorus, serum calcium, TSH, FT4, and CKD.

Bold values indicate statistically significant results (*P* < .05).

ALP = alkaline phosphatase, BMD = bone mineral density, BMI, body mass index, CI = confidence interval, CKD = chronic kidney disease, FT4 = free thyroxine, Hb = hemoglobin, PIR = poverty-to-income ratio, TGAB = thyroglobulin antibody, TPOAB = thyroid peroxidase antibody, TSH = thyroid-stimulating hormone.

**Table 2:** Association between TPOAB and TGAB titers with BMD.

Femoral neck BMD		Model 1: β (95% CI)	*P*	Model 2: β (95% CI)	*P*	Model 3: β (95% CI)	*P*
TGAB	Negative	Reference		Reference		Reference	
	Low-titer Group	−0.041 (−0.072, −0.010)	**.010**	−0.018 (−0.043, 0.006)	.132	−0.016 (−0.041, 0.008)	.182
	High-titer Group	−0.058 (−0.100, −0.017)	**.008**	−0.017 (−0.074, 0.040)	.532	−0.029 (−0.096, 0.038)	.368
	*P* for trend		**.001**		.551		.587
TPOAB	Negative	Reference		Reference		Reference	
	Low-titer group	−0.061 (−0.077, −0.045)	**<.0001**	−0.027 (−0.045, 0.009)	.124	−0.026 (−0.044, 0.008)	.132
	High-titer group	−0.12 (−0.158, −0.083)	**<.0001**	−0.086 (−0.115, −0.057)	**<.001**	−0.085 (−0.115, −0.056)	**.002**
	*P* for trend		**<.0001**		**.011**		**.006**
Lumbar spine BMD		Model 1: β (95% CI)	*P*	Model 2: β (95% CI)	*P*	Model 3: β (95% CI)	*P*
TGAB	Negative	Reference		Reference		Reference	
	Low-titer group	−0.064 (−0.104, 0.032)	.082	0.021 (−0.042, 0.027)	.227	0.011 (−0.036, 0.041)	.313
	High-titer group	−0.043 (−0.104, 0.02)	.192	−0.026 (−0.065, 0.013)	.183	−0.034 (−0.076, 0.009)	.115
	*P* for trend		.421		.292		.281
TPOAB	Negative	Reference		Reference		Reference	
	Low-titer group	−0.056 (−0.074, 0.038)	.162	−0.035 (−0.053, 0.017)	.233	−0.034 (−0.054, 0.014)	.245
	High-titer group	−0.076 (−0.130, −0.023)	**<.001**	−0.051 (−0.097, −0.005)	**<.001**	−0.053 (−0.099, −0.007)	**.004**
	*P* for trend		**<.001**		**<.001**		**.002**

Values are β coefficients and 95% CIs for BMD. Antibody titers were categorized into negative, low-titer, and high-titer groups, with the negative group as the reference. Model 1: adjusted for none. Model 2: adjusted for age, gender, race, PIR, BMI, alcohol consumption, smoking status, and physical activity. Model 3 was additionally adjusted for Hb, dietary vitamin D intake, ALP, serum phosphorus, serum calcium, TSH, FT4, and CKD.

Bold values indicate statistically significant results (*P* < .05).

ALP = alkaline phosphatase, BMD = bone mineral density, BMI, body mass index, CI = confidence interval, CKD = chronic kidney disease, FT4 = free thyroxine, Hb = hemoglobin, PIR = poverty-to-income ratio, TGAB = thyroglobulin antibody, TPOAB = thyroid peroxidase antibody, TSH = thyroid-stimulating hormone.

When antibody titers were further analyzed (Table 2), no significant association was observed between TGAB titers and BMD after full adjustment (*P* > .05). In contrast, elevated TPOAB titers were independently associated with decreased BMD at both sites. Compared with the TPOAB-negative group, participants with high TPOAB titers had significantly reduced femoral neck BMD (model 3: β = −0.085 [95% CI: −0.115 to −0.056], *P* = .002) and lumbar spine BMD (model 3: β = −0.053 [95% CI: −0.099 to −0.007], *P* = .004), with a significant linear trend across titer categories (*P* for trend < .05). Taken together, these findings indicate that thyroid autoantibody positivity, especially double positivity for TPOAB and TGAB and higher TPOAB titers, are independently associated with lower BMD.

**Figure 1.**
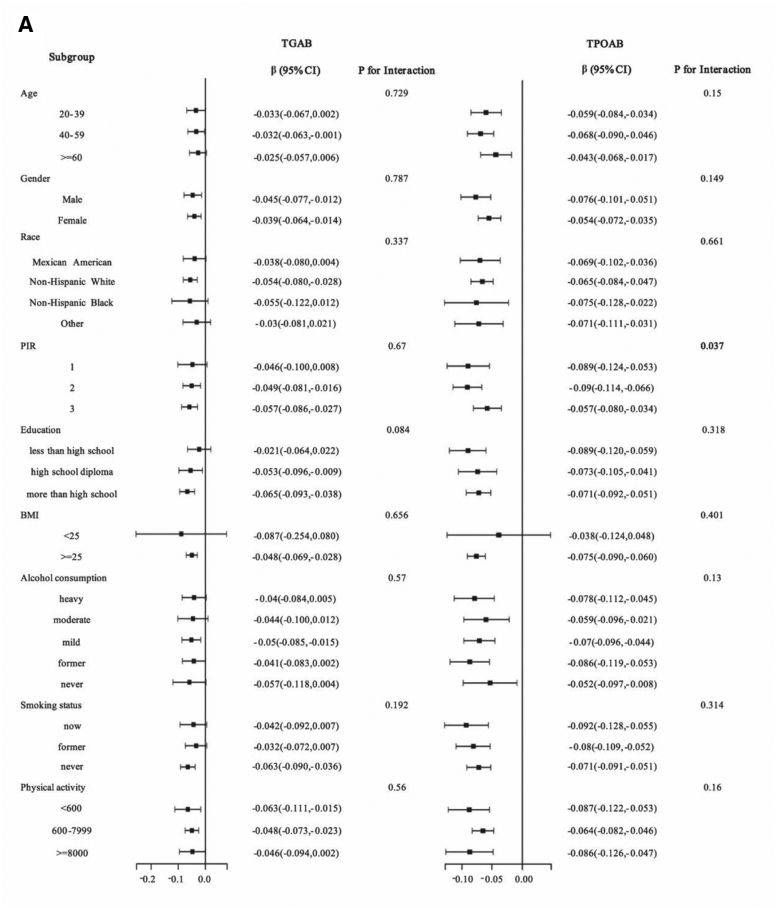
Subgroup analyses of the associations between thyroid autoantibodies and BMD. Forest plots showing subgroup analyses of the associations of TGAB and TPOAB with BMD. Panel (A) shows the associations with femoral neck BMD, and panel (B) shows the associations with lumbar spine BMD. Subgroups were stratified by age, sex, race, PIR, educational level, BMI, alcohol consumption, smoking status, and physical activity. Points represent regression coefficients (β), and horizontal lines represent 95% CIs. *P* values for interaction were calculated to evaluate whether the associations differed across subgroups. BMD = bone mineral density, BMI, body mass index, CI = confidence interval, PIR = poverty-to-income ratio, TGAB = thyroglobulin antibody, TPOAB = thyroid peroxidase antibody.

Subgroup analyses were performed by age, sex, race, PIR, education, BMI, alcohol consumption, smoking, and PA (Fig. 1). Overall, TPOAB and TGAB were inversely correlated with femoral neck and lumbar spine BMD, and the association between TPOAB and lumbar spine BMD was statistically more significant. The inverse association was more pronounced among older adults (*P* for interaction = .021), those with lower education (*P* for interaction = .018), insufficient physical activity (*P* for interaction = .017), and alcohol consumption (*P* for interaction = .043). TGAB showed weaker and less consistent patterns, with a significant interaction detected only across alcohol strata. Overall, TPOAB positivity was linked to reduced bone mass, particularly in individuals with adverse social and lifestyle factors, with stronger evidence at the lumbar spine.

### 3.3. Associations of TPOAB and TGAB with fracture risk in NHANES

To further elucidate the association between thyroid autoantibodies and fracture risk, fracture history at different skeletal sites was extracted and evaluated using multivariable logistic regression models. As shown in Table 3, after adjusting for potential confounding factors, only TPOAB positivity was significantly associated with an increased risk of spinal fracture (model 3: OR = 2.87 [95% CI: 1.02–8.12], *P* = .04). Stratified analysis by antibody titer further revealed that participants with high TPOAB titers had a significantly higher risk of vertebral fracture (model 3: OR = 8.60 [95% CI: 1.03–71.52], *P* = .04). The number of spinal fracture cases in each antibody titer stratum is presented in the table. Although high-titer TPOAB was associated with an increased risk of spinal fracture, the number of fracture events was small in some strata, leading to wide confidence intervals. Thus, this result is presented as an exploratory finding and should be interpreted cautiously. A significant linear trend was also observed with increasing antibody titers (*P* for trend = .01), suggesting a potential dose-response relationship between TPOAB levels and spinal fracture risk. In contrast, TGAB positivity and titer levels were not significantly associated with spinal fracture, with no statistically significant differences observed across all models (*P* > .05). Furthermore, analyses of wrist and hip fractures (Table S2, Supplemental Digital Content 2) revealed no significant associations between either TPOAB or TGAB (all *P* > .05). Collectively, elevated TPOAB levels were independently associated with a higher risk of spinal fracture, whereas no significant associations were observed between thyroid autoantibodies and the risk of wrist or hip fractures. These findings suggest that the correlation between increased TPOAB levels and fracture risk may be site-specific.

**Table 3:** Association between TPOAB and TGAB with spinal fracture.

Antibody Status			Model 1: OR (95% CI)	*P*	Model 2: OR (95% CI)	*P*	Model 3: OR (95% CI)	*P*
TGAB (−)			Reference		Reference		Reference	
TGAB (+)			1.23 (0.34, 4.52)	.74	1.13 (0.28, 4.51)	.86	1.28 (0.30, 5.38)	.72
TPOAB (−)			Reference		Reference		Reference	
TPOAB (+)			3.12 (0.87, 11.16)	.08	2.85 (1.01, 8.07)	**.05**	2.87 (1.02, 8.12)	**.04**
TGAB&TPOAB (−)			Reference		Reference		Reference	
TGAB&TPOAB (+)			1.81 (0.45, 7.24)	.39	1.67 (0.38, 7.46)	.48	1.90 (0.42, 8.66)	.37
Antibody		Titer level, cases	Model 1: OR (95% CI)	*P*	Model 2: OR (95% CI)	*P*	Model 3: OR (95% CI)	*P*
TGAB	Negative, 88/2799		Reference		Reference		Reference	
	Low-titer group, 42/191		1.44 (0.39, 5.29)	.57	1.34 (0.33, 5.39)	.67	1.51 (0.35, 6.45)	.55
	High-titer group, 34/104		1.51 (0.45, 5.31)	.51	1.87 (0.51, 6.01)	.45	2.01 (0.78, 7.89)	.65
	*P* for trend			.8		.93		.8
TPOAB	Negative, 65/2633		Reference		Reference		Reference	
	Low-titer group, 42/388		2.76 (0.62, 12.27)	.17	2.46 (0.64, 9,42)	.17	2.88 (0.80, 10.30)	.10
	High-titer group, 22/73		8.52 (1.53, 47.37)	**.02**	9.12 (1.17, 70.83)	**.04**	8.60 (1.03, 71.52)	**.04**
	*P* for trend			**.02**		**.02**		**.01**

Values are ORs and 95% CIs for spinal fracture. The numbers in parentheses indicate spinal fracture cases/total participants within each antibody-titer stratum. Model 1 was unadjusted. Model 2 was adjusted for age, gender, race, PIR, BMI, alcohol consumption, smoking status, and physical activity. Model 3 was additionally adjusted for Hb, dietary vitamin D intake, ALP, serum phosphorus, serum calcium, TSH, FT4, and CKD.

Bold values indicate statistically significant results (*P* < .05).

ALP = alkaline phosphatase, BMD = bone mineral density, BMI, body mass index, CI = confidence interval, CKD = chronic kidney disease, FT4 = free thyroxine, Hb = hemoglobin, OR = odds ratio, PIR = poverty-to-income ratio, TGAB = thyroglobulin antibody, TPOAB = thyroid peroxidase antibody, TSH = thyroid-stimulating hormone.

**Figure 2.**
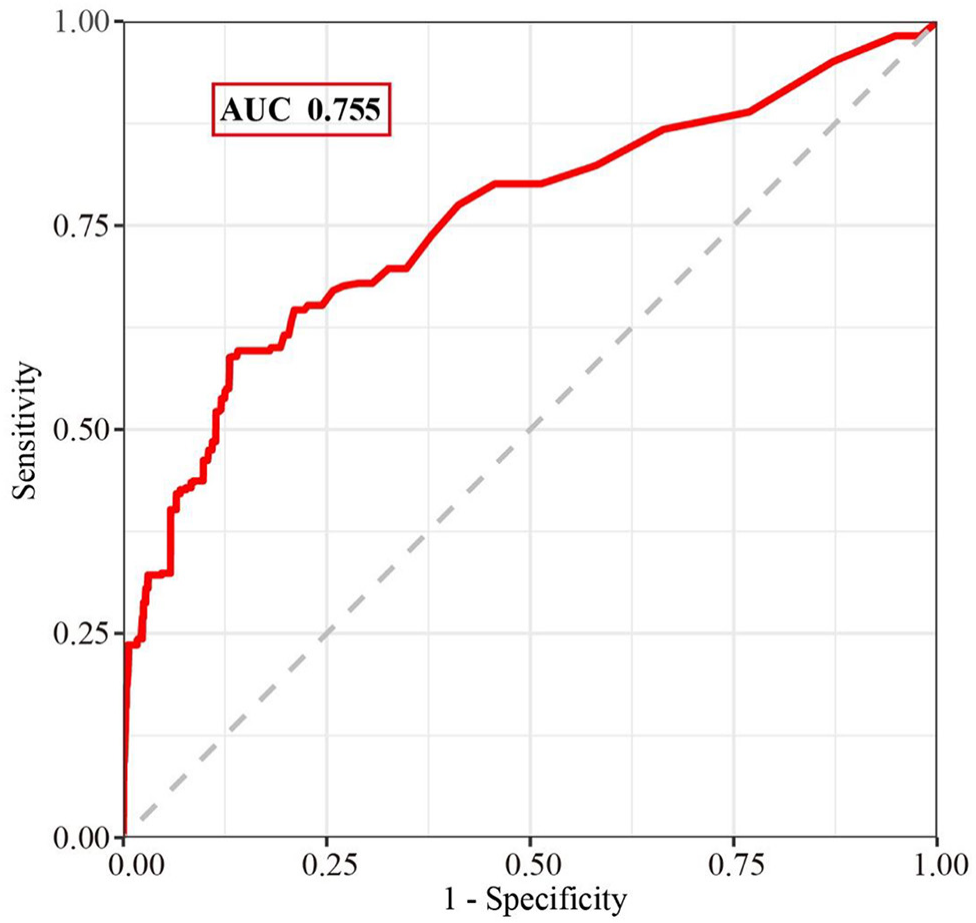
ROC curve for TPOAB titers in discriminating spinal fracture. The ROC curve shows the discriminatory ability of TPOAB titers for spinal fracture. TPOAB titers showed moderate discriminatory performance for spinal fracture, with an AUC of 0.755. AUC = area under the curve, ROC = receiver operating characteristic, TPOAB = thyroid peroxidase antibody.

In addition, we performed an exploratory ROC curve analysis to evaluate the discriminatory ability of TPOAB titers for spinal fracture (Fig. 2). In the analysis, TPOAB titers showed moderate discrimination for spinal fracture, with an area under the curve (AUC) of 0.755. This finding is consistent with the observed association between high-titer TPOAB and spinal fracture risk. Nevertheless, given the cross-sectional design, limited fracture events, and lack of external validation, the ROC result should be considered hypothesis-generating rather than evidence supporting clinical risk prediction.

### 3.4. Genetic evidence for the association between HT and OP in MR

**Figure 3.**
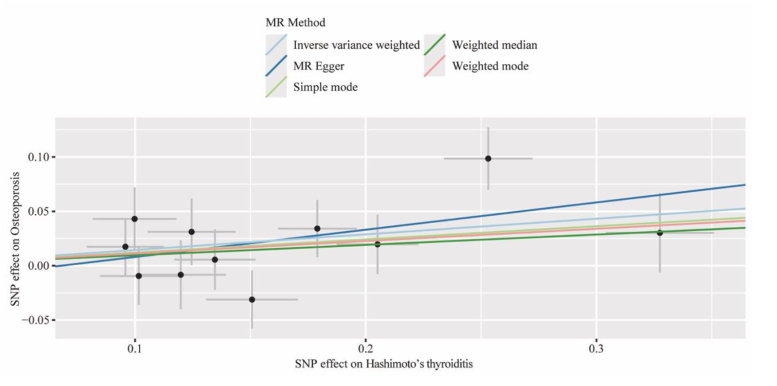
Scatter plot of the MR analysis for the effect of HT on OP. A scatter plot showing the genetic associations of SNPs with HT and OP. Each point represents an individual SNP used as an instrumental variable. The *x*-axis represents the SNP effect on HT, and the *y*-axis represents the SNP effect on OP. The slopes of the fitted lines indicate the causal effect estimates obtained using different MR methods, including IVW, MR-Egger, weighted median, weighted mode, and simple mode methods. HT = Hashimoto’s thyroiditis, IVW, inverse-variance weighted, MR = Mendelian randomization, OP = osteoporosis, SNP = single-nucleotide polymorphism.

**Figure 4.**
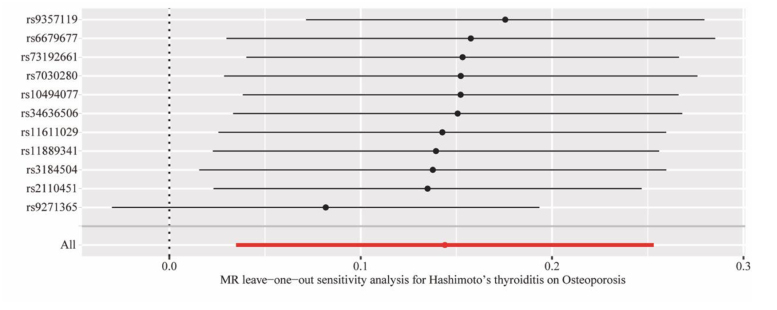
Leave-one-out sensitivity analysis of the MR estimate for the effect of HT on OP. A leave-one-out analysis assessing whether the MR estimate for the association between genetically predicted HT and OP was driven by any single instrumental variable. Each row shows the causal estimate after excluding 1 SNP at a time. The “All” row represents the overall estimate using all included SNPs. The consistency of the estimates after sequential SNP exclusion suggests that the result was not primarily driven by any individual genetic variant. HT = Hashimoto’s thyroiditis, MR = Mendelian randomization, OP = osteoporosis, SNP = single-nucleotide polymorphism.

Given the significant inverse associations observed in the multivariable regression analyses, we proceeded with MR analyses to infer the causal effect of HT on OP risk. As shown in Table 4, using the IVW method, HT was positively associated with OP risk (OR = 1.155 [95% CI: 1.036–1.288], *P* = .010). Moreover, the direction of the causal estimate and the magnitude of the effect were consistent across complementary MR methods, supporting the reliability of the finding. Figure 3 is a scatter plot that intuitively demonstrates consistent overall trends across different MR methods. Cochran *Q* test indicated no significant heterogeneity (IVW: *Q* = 11.978, *P* = .287; MR-Egger: *Q* = 11.267, *P* = .258). To assess whether SNPs might influence OP through alternative pathways, we further evaluated horizontal pleiotropy. No evidence of pleiotropy was detected using the MR-Egger intercept test (*P* = .471) or the MR-PRESSO global test (*P* = .261), and no outlier SNPs were identified. Leave-one-out sensitivity analysis (Fig. 4) confirmed that the causal association between HT and OP was not driven by any single SNP. Collectively, genetic analyses suggest that genetic liability to HT exerts a potential causal effect on clinically diagnosed OP.

**Table 4:** MR estimates from each method of assessing the causal effect of HT on OP.

Method	β	SE	OR (95% CI)	*P*	Heterogeneity test	MR-Egger intercept
IVW	0.144	0.056	1.155 (1.036,1.288)	**.010**	Q = 11.978, *P* = .287	
MR-Egger	0.251	0.153	1.285 (0.952,1.734)	.135	Q = 11.267, *P* = .258	*P* = .471
Weighted median	0.095	0.071	1.100 (0.953,1.270)	.215		
Simple mode	0.121	0.120	1.128 (0.893,1.426)	.363		
Weighted mode	0.113	0.107	1.120 (0.903,1.389)	.337		
*P* value for MR-PRESSO global test = .261						

MR estimates are presented as β coefficients, ORs, 95% CIs, and *P* values for the effect of genetically predicted HT on OP. The IVW method was used as the primary analysis, with MR-Egger, weighted median, simple mode, and weighted mode methods used as sensitivity analyses. Cochran *Q* test assessed heterogeneity, and the MR-Egger intercept and MR-PRESSO global tests assessed horizontal pleiotropy. ORs were derived by exponentiating β coefficients.

Bold values indicate statistically significant results (*P* < .05).

CI = confidence interval, HT = Hashimoto’s thyroiditis, IVW, inverse-variance weighted, MR = Mendelian randomization, MR-PRESSO = Mendelian Randomization Pleiotropy RESidual Sum and Outlier, OP = osteoporosis, OR = odds ratio, SNP = single-nucleotide polymorphism.

To evaluate potential reverse causation, we conducted MR analyses with OP as the exposure and HT as the outcome (Table S3, Supplemental Digital Content 3 and Figs. S1 and S2, Supplemental Digital Content 4). The IVW method yielded *P* > .05, we found no evidence that genetic liability to OP increases the risk of HT.

## 4. Discussion

In this study, we combined data from the nationally representative NHANES 2007–2010 cohort with 2-sample MR analyses to examine the relationship between HT and OP. After adjusting for demographic, lifestyle, and biochemical confounders, positive status and high titers of TPOAB were negatively correlated with BMD at the femoral neck and lumbar spine. A clear dose-response relationship was also observed: higher TPOAB titers corresponded to more pronounced bone loss. We detected an association between TPOAB and spinal fracture, whereas no significant correlation was found for wrist or hip fracture. Such site-specific differences may be attributed to variations in skeletal structure. Furthermore, exploratory ROC analysis further indicated that TPOAB titers had a moderate discriminatory ability for spinal fracture. However, the estimation precision of this result was limited due to the small number of fracture events; thus, this finding only serves as a hypothesis for subsequent research. Spinal fractures are often detected incidentally during imaging examinations. In addition, individuals with thyroid autoimmunity receive medical surveillance at varying frequencies, which may influence the observed associations and introduce potential detection bias. In conclusion, thyroid autoimmunity, particularly TPOAB positivity, may contribute to bone metabolism disorders independent of overt thyroid dysfunction and is correlated with adverse skeletal outcomes. Further studies are still required to determine whether TPOAB can be independently incorporated into fracture risk prediction models.

MR analyses provided supplementary genetic evidence for a potential causal association between genetic liability to HT and an increased risk of clinically diagnosed OP. However, we could not confirm that TPOAB titers act as direct causal mediators for this relationship. Overall, thyroid autoimmunity appears to be involved in the progression of skeletal damage, though whether thyroid autoantibodies themselves are direct pathogenic factors remains unclear.

In recent years, the crosstalk between the skeletal and immune systems has emerged as an important research field. Immune cells originate from the bone marrow and develop alongside osteoclast precursors within the same microenvironment.^[12,13]^ Aberrant activation of the immune system triggers a robust release of proinflammatory cytokines and chemokines. Cytokines such as IL-1β, IL-17, and IL-23 are key contributors to HT pathogenesis,^[21]^ and chronic thyroid autoimmunity–related inflammation may increase the RANKL/OPG ratio via IL-6 and TNF-α signaling, thereby enhancing osteoclastic activity, suppressing osteoblastic function, and promoting bone resorption and loss.^[22]^ Thyroid autoantibody titers may therefore reflect the intensity and persistence of autoimmune activation. In our study, higher TPOAB titers were associated with lower BMD and higher spinal fracture risk, suggesting that antibody titers serve as an indirect marker of immune activation and its impact on bone metabolism. Elevated titers may indicate stronger inflammatory responses that aggravate bone loss through inflammation-mediated osteoclastic pathways. MR analyses provided additional genetic evidence indicating that genetic liability to clinically diagnosed HT was associated with an increased risk of OP. Notably, MR analyses adopted genetic predisposition to clinically diagnosed HT as the exposure, while observational studies used peripheral thyroid autoantibody titers to reflect autoimmune activity. Although the 2 indicators are correlated, they are not entirely equivalent. Therefore, the MR results serve only as supporting evidence and cannot directly confirm a causal effect of antibody titers on bone loss.

Among thyroid autoantibodies, TPOAB is regarded as a stable marker of autoimmune activity. It is mainly composed of IgG1 and IgG4 subclasses that bind complement and induce thyroid tissue damage through complement-dependent cytotoxicity (CDC) and antibody-dependent cell-mediated cytotoxicity (ADCC), accompanied by inflammatory cytokine release and remodeling of the immune microenvironment.^[13]^ In this study, we observed a dose-dependent relationship between TPOAB titers and reduced BMD. High titers likely indicate stronger and more persistent immune attacks, enhancing osteoclast activity and accelerating bone resorption. Collectively, TPOAB levels can not only indicate the presence of disease but also reflect the intensity of autoimmune responses and their impacts on bone metabolism. Thus, TPOAB may serve as an indirect biomarker for adverse skeletal outcomes. Previous studies have shown that euthyroid patients with HT have lower lumbar spine and femoral neck BMD than individuals without HT, and that OP prevalence increases with disease duration, supporting long-term adverse effects of sustained autoimmunity on bone health.

Poubelle et al^[23]^ proposed that neutrophils participate in bone remodeling within inflammatory microenvironments. They demonstrated abundant neutrophils at inflammatory sites and showed that, under certain conditions, neutrophils can directly contribute to adaptive immunity and bone remodeling through RANK (receptor activator of nuclear factor-κB) expression. Other evidence indicates that the neutrophil-to-lymphocyte ratio (NLR) has diagnostic value for HT.^[24]^ As a systemic inflammatory marker, NLR reflects both increased neutrophils and/or decreased lymphocytes. The distinctive role of neutrophils described by Poubelle et al may partly explain how elevated NLR in HT contributes to impaired bone metabolism and reduced bone density.^[23]^ Based on our findings, we speculate that elevated TPOAB titers may be closely linked to increased NLR, with both reflecting the intensity and persistence of immune activation. High antibody titers may promote complement activation and immune cell recruitment, further driving proinflammatory neutrophil activation. This self-perpetuating inflammatory loop could be a key pathway connecting HT-related immune activity to abnormal bone metabolism.^[11]^ Accordingly, elevated NLR together with high TPOAB titers in HT may capture not only disease activity and immune burden but also the detrimental impact of chronic inflammation on bone formation–resorption balance, warranting longitudinal studies to clarify their combined predictive value for bone metabolic abnormalities and fracture risk.

Previous studies have reported that TPOAB positivity is associated with lower BMD in postmenopausal women.^[14]^ Our findings corroborate and extend this association, showing that TPOAB positivity and higher titers are linked to reduced BMD independent of thyroid hormone levels, suggesting that thyroid autoimmunity affects bone metabolism beyond hormonal alterations. We also observed a dose-response pattern; higher TPOAB titers were associated with greater BMD loss and higher spinal fracture risk, especially in older adults and those with adverse socioeconomic or behavioral factors. MR analyses provided genetic evidence suggesting a potential causal association between genetic liability to HT and elevated OP risk. Our findings indicate that thyroid autoimmunity may be involved in the progression of skeletal damage. Nevertheless, based on the data from this observational study, the specific role of antibody-mediated mechanisms remains unclear. Clinically, TPOAB positivity and elevated titers may serve as potential biomarkers for the early screening of individuals at high risk of low bone mass or osteoporotic fractures. Nevertheless, our findings do not support the use of isolated TPOAB titers as an independent criterion for initiating pharmacological intervention against OP. Instead, our study highlights the need for prospective studies to explore whether persistently elevated TPOAB titers can further optimize current OP risk stratification when integrated with established bone risk indicators.

Currently, however, there are no effective therapies targeting early immune dysregulation in HT. Selenium (Se) shows immunomodulatory effects: Se supplementation in HT can reduce TPOAB and TGAB titers, enhance glutathione-peroxidase-3 activity, increase activated regulatory T (Treg) cells, decrease IFN-γ, and increase IL-1β.^[25,26]^ By improving antioxidant capacity and upregulating activated Treg cells, Se supplementation benefits thyroid autoantibody levels and thyroid function.^[25]^ With advancing age, serum Se levels are negatively associated with bone turnover markers and positively associated with BMD.^[27]^ A US population-based study also found that higher Se status was linked to lower FRAX (Fracture Risk Assessment Tool) scores and reduced fracture history.^[28]^ Importantly, decreased antibody titers may indicate the immunologic benefit of Se therapy. Although previous studies have confirmed that Se supplementation can reduce thyroid autoantibody titers in certain settings, it remains unclear whether such declines in antibody levels translate to improved BMD and reduced fracture risk. Before Se supplementation or other antibody-lowering regimens are applied for skeletal protection, randomized controlled trials with bone-related outcomes as primary endpoints are warranted for verification.

This study has several strengths. We evaluated both thyroid autoantibody positivity and antibody titers, allowing us to identify a dose-dependent association between TPOAB levels, lower BMD, and spinal fracture risk. In addition, the combination of observational analyses and MR provided complementary evidence and strengthened the interpretation of our findings. Several limitations should also be noted. First, the NHANES data were cross-sectional, and thyroid antibodies and other laboratory markers were measured only once, preventing assessment of long-term autoimmune exposure or disease duration. Second, only euthyroid individuals were included; although this reduced confounding by overt thyroid dysfunction, the findings may not apply to patients with subclinical hypothyroidism or those receiving levothyroxine. Third, the 600 IU/mL cutoff for high antibody titers was based on the upper reporting limit of our institutional laboratory. Given the lack of universally accepted thyroid antibody cutoffs for stratifying skeletal risk, this grouping approach was adopted merely as an exploratory standard for clinical interpretation. Fourth, the MR analysis was based mainly on European ancestry GWAS and FinnGen data, whereas the observational analysis used a multiethnic US sample, which may introduce population heterogeneity. Finally, OP in FinnGen was defined using ICD codes and may primarily reflect clinically diagnosed moderate-to-severe cases. Therefore, our findings do not support using TPOAB titers alone as a criterion for initiating pharmacological treatment for osteoporosis. Future prospective studies are needed to determine whether persistent TPOAB elevation improves fracture-risk prediction beyond established clinical risk factors.

From a clinical perspective, our findings suggest that thyroid autoimmunity may be an under-recognized factor contributing to impaired bone health. Routine screening of thyroid autoantibodies in all patients with OP is not recommended based solely on our findings. Nevertheless, for patients with unexplained reduced BMD or fractures after the exclusion of other secondary causes, clinicians may consider screening for thyroid autoimmune abnormalities. This multidimensional cross-sectional analysis supports the overall involvement of thyroid autoimmunity in the progression of skeletal damage but cannot confirm that TPOAB elevation directly causes bone loss. Further prospective and interventional studies are therefore required to determine whether targeted assessment and intervention for thyroid autoimmunity can optimize long-term skeletal prognosis.

## 5. Conclusions

In this study, measured TPOAB titers were associated with lower BMD and higher spinal fracture risk among euthyroid individuals, whereas genetic liability to HT was associated with increased OP risk in MR analyses. These findings provide complementary evidence that thyroid autoimmunity may be involved in skeletal deterioration. However, the observational and genetic analyses address distinct but related questions, and the MR results should not be interpreted as proof that antibody titers themselves are causally responsible for bone loss. The specific contribution of antibody-mediated mechanisms versus other aspects of the autoimmune process remains to be elucidated in longitudinal studies and mechanistic investigations.

## Acknowledgments

We extend our gratitude to all the investigators and participants of NHANES, FinnGen, and the GWAS Catalog for making their data publicly available.

## Author contributions

**Conceptualization:** Rujie Jiang, Fang Wang.

**Data curation:** Rujie Jiang, Shuyu Yu, Xiaowen Zhong.

**Formal analysis:** Rujie Jiang, Jie You.

**Methodology:** Fang Wang.

**Resources:** Juntao Liu, Chuan Wan.

**Software:** Rujie Jiang.

**Supervision:** Jiapeng Niu, Fang Wang.

**Visualization:** Xiaochao Xie, Yanan Sun.

**Writing – original draft:** Rujie Jiang, Shuyu Yu.

**Writing – review & editing:** Rujie Jiang, Jiapeng Niu, Fang Wang.










