# Supplementary material for: High titers of thyroid peroxidase antibodies as a potential risk factor for osteoporosis: A cross-sectional NHANES study and bidirectional Mendelian randomization analysis
Source: Medicine (Baltimore). 2026 Jul 24;105(30):e49917. doi: 10.1097/MD.0000000000049917 (PMC13406175; doi:10.1097/MD.0000000000049917)
Supplement: Supplementary file 1 [file medi-105-e49917-s001.docx]

**Supplementary Table 1. Clinical and Laboratory characteristics of participants**

| **Variable** | **Femoral Neck BMD** | | | | | **Lumbar Spine BMD** | | | | |
| --- | --- | --- | --- | --- | --- | --- | --- | --- | --- | --- |
|  | | Q1 | Q2 | Q3 | P | | Q1 | Q2 | Q3 | P |
| Age, y | | 49.98(0.71) | 41.50(0.53) | 36.06(0.57) | **< 0.0001** | | 45.97(0.72) | 40.45(0.68) | 40.72(0.65) | **< 0.0001** |
| Gender | |  |  |  | **< 0.0001** | |  |  |  | **< 0.001** |
| Female | | 600(63.03) | 470(44.75) | 342(32.34) |  | | 554(52.77) | 453(47.33) | 405(39.18) |  |
| Male | | 436(36.97) | 562(55.25) | 684(67.66) |  | | 484(47.23) | 572(52.67) | 626(60.82) |  |
| Race | |  |  |  | **< 0.0001** | |  |  |  | **< 0.0001** |
| Mexican American | | 181(7.11) | 195(8.00) | 185(9.50) |  | | 235(9.60) | 191(9.75) | 135(5.50) |  |
| Non-Hispanic Black | | 92(3.68) | 149(7.63) | 271(15.85) |  | | 85(3.70) | 168(9.77) | 259(13.70) |  |
| Non-Hispanic White | | 614(77.43) | 544(76.40) | 422(63.92) |  | | 559(74.38) | 517(71.85) | 504(71.32) |  |
| Other | | 149(11.78) | 144(7.97) | 148(10.72) |  | | 159(12.32) | 149(8.62) | 133(9.48) |  |
| PIR | |  |  |  | 0.24 | |  |  |  | 0.83 |
| 1 | | 185(10.50) | 207(14.23) | 206(14.88) |  | | 216(13.22) | 202(13.77) | 180(12.88) |  |
| 2 | | 440(33.95) | 424(32.68) | 407(31.86) |  | | 441(34.37) | 406(31.59) | 424(32.52) |  |
| 3 | | 411(55.55) | 401(53.09) | 413(53.26) |  | | 381(52.41) | 417(54.64) | 427(54.60) |  |
| Education | |  |  |  | **0.05** | |  |  |  | 0.12 |
| high school diploma | | 240(21.44) | 246(24.60) | 233(22.71) |  | | 260(24.04) | 211(20.43) | 248(24.43) |  |
| less than high school | | 272(15.90) | 232(13.71) | 279(19.74) |  | | 288(16.82) | 274(18.29) | 221(14.31) |  |
| more than high school | | 524(62.66) | 554(61.69) | 514(57.55) |  | | 490(59.14) | 540(61.28) | 562(61.25) |  |
| BMI, kg/m2 | | 25.63(0.20) | 27.32(0.17) | 29.47(0.21) | **< 0.0001** | | 25.93(0.25) | 27.57(0.20) | 28.95(0.16) | **< 0.0001** |
| Alcohol consumption | |  |  |  | **< 0.0001** | |  |  |  | **0.01** |
| former | | 225(18.25) | 153(13.14) | 112(7.82) |  | | 210(16.66) | 136(10.75) | 144(11.59) |  |
| heavy | | 162(17.46) | 286(27.35) | 324(33.55) |  | | 223(21.83) | 261(26.25) | 288(30.66) |  |
| mild | | 334(35.42) | 326(35.26) | 309(32.88) |  | | 312(35.81) | 322(33.91) | 335(33.86) |  |
| moderate | | 162(18.33) | 161(16.85) | 182(18.12) |  | | 148(15.15) | 190(21.02) | 167(16.98) |  |
| never | | 153(10.54) | 106(7.40) | 99(7.63) |  | | 145(10.55) | 116(8.07) | 97(6.91) |  |
| Smoking status | |  |  |  | **0.04** | |  |  |  | 0.86 |
| former | | 275(25.81) | 239(22.60) | 200(18.69) |  | | 241(23.07) | 231(22.62) | 242(21.21) |  |
| never | | 531(52.55) | 536(51.83) | 576(59.33) |  | | 556(53.04) | 542(54.54) | 545(56.11) |  |
| now | | 230(21.64) | 257(25.56) | 250(21.99) |  | | 241(23.89) | 252(22.84) | 244(22.68) |  |

| **Variable** | **Femoral Neck BMD** | | | | | **Lumbar Spine BMD** | | | | |
| --- | --- | --- | --- | --- | --- | --- | --- | --- | --- | --- |
| Physical activity, MET min/week |  |  |  | **< 0.001** |  | |  |  | **0.03** |  |
| <600 | 215(19.21) | 157(13.32) | 127(11.94) |  | 201(17.56) | | 153(13.17) | 145(13.53) |  |  |
| 600-7999 | 655(66.93) | 640(64.26) | 621(65.03) |  | 641(65.12) | | 642(67.56) | 633(63.44) |  |  |
| >=8000 | 166(13.86) | 235(22.42) | 278(23.03) |  | 196(17.33) | | 230(19.27) | 253(23.03) |  |  |
| Hb, g/dL | 14.14(0.09) | 14.55(0.08) | 14.74(0.07) | **< 0.0001** | 14.45(0.08) | | 14.46(0.08) | 14.55(0.08) | 0.45 |  |
| Vitamin D intake, µg/d | 4.35(0.16) | 5.13(0.25) | 5.26(0.26) | **0.02** | 4.75(0.18) | | 4.74(0.19) | 5.28(0.21) | **0.05** |  |
| ALP, U/L | 66.96(0.71) | 64.35(0.92) | 64.47(1.04) | **0.02** | 67.95(0.92) | | 64.54(0.69) | 63.32(0.79) | **0.002** |  |
| Serum phosphorus, mg/dL | 3.77(0.02) | 3.71(0.02) | 3.73(0.02) | **0.04** | 3.77(0.03) | | 3.74(0.02) | 3.69(0.03) | 0.12 |  |
| Serum calcium, mg/dL | 9.45(0.02) | 9.43(0.02) | 9.44(0.03) | 0.65 | 9.47(0.02) | | 9.44(0.02) | 9.39(0.03) | **0.01** |  |
| CRP, mg/dL | 0.32(0.03) | 0.30(0.01) | 0.35(0.02) | 0.1 | 0.30(0.03) | | 0.31(0.02) | 0.35(0.02) | 0.26 |  |
| TSH, mIU/mL | 2.02(0.05) | 2.19(0.44) | 1.88(0.06) | 0.22 | 1.94(0.06) | | 2.29(0.46) | 1.87(0.05) | 0.37 |  |
| FT4, ng/dL | 0.79(0.01) | 0.77(0.01) | 0.78(0.01) | 0.12 | 0.79(0.01) | | 0.78(0.01) | 0.78(0.01) | 0.28 |  |
| DM |  |  |  | 0.21 |  | |  |  | 0.11 |  |
| No | 889(89.91) | 909(92.45) | 914(92.21) |  | 913(91.39) | | 904(93.38) | 895(90.00) |  |  |
| Yes | 147(10.09) | 123(7.55) | 112(7.79) |  | 125(8.61) | | 121(6.62) | 136(10.00) |  |  |
| CKD |  |  |  |  |  | |  |  |  |  |
| No | 869(89.27) | 940(93.70) | 941(94.44) |  | 890(90.07) | | 940(94.54) | 920(92.96) |  |  |
| Yes | 167(10.73) | 92(6.30) | 85(5.56) |  | 148(9.93) | | 85(5.46) | 111(7.04) |  |  |
| TGAB, IU/mL | 11.48(4.17) | 11.03(5.07) | 5.67(1.73) | 0.09 | 14.51(5.56) | | 7.86(4.86) | 6.02(1.34) | 0.3 |  |
| TPOAB, IU/mL | 36.24(5.36) | 19.91(3.75) | 13.7(2.46) | **<0.001** | 29.60(3.87) | | 20.91(5.85) | 18.72(2.75) | **0.03** |  |
| TGAB |  |  |  | **0.001** |  | |  |  | **< 0.001** |  |
| Negative | 929(90.13) | 966(93.79) | 976(95.65) |  | 920(89.25) | | 972(94.87) | 979(95.46) |  |  |
| Positive | 107(9.87) | 66(6.21) | 50(4.35) |  | 118(10.75) | | 53(5.13) | 52(4.54) |  |  |
| TPOAB |  |  |  | **< 0.0001** |  | |  |  | **< 0.0001** |  |
| Negative | 801(76.87) | 915(89.39) | 948(91.45) |  | 795(78.00) | | 930(89.14) | 939(90.85) |  |  |
| Positive | 235(23.13) | 117(10.61) | 78(8.55) |  | 243(22.00) | | 95(10.86) | 92(9.15) |  |  |
| Wrist fracture |  |  |  | 0.43 |  | |  |  | **0.05** |  |
| No | 919(88.67) | 933(90.01) | 948(90.64) |  | 914(87.68) | | 938(89.80) | 948(91.78) |  |  |
| Yes | 117(11.33) | 99(9.99) | 78(9.36) |  | 124(12.32) | | 87(10.20) | 83(8.22) |  |  |

| **Variable** | **Femoral Neck BMD** | | | | | | **Lumbar Spine BMD** | | | |
| --- | --- | --- | --- | --- | --- | --- | --- | --- | --- | --- |
| Spine fracture | |  |  |  | **0.01** |  | |  |  | 0.35 |
| No | | 1013(97.24) | 1019(99.05) | 1015(98.83) |  | 1019(97.87) | | 1013(99.08) | 1015(98.25) |  |
| Yes | | 23(2.76) | 13(0.95) | 11(1.17) |  | 19(2.13) | | 12(0.92) | 16(1.75) |  |
| Hip fracture | |  |  |  | 0.09 |  | |  |  | 0.83 |
| No | | 1020(97.90) | 1025(99.59) | 1020(98.97) |  | 1028(98.60) | | 1015(98.98) | 1023(98.95) |  |
| Yes | | 16(2.10) | 5(0.41) | 6(1.03) |  | 9(1.40) | | 10(1.02) | 8(1.05) |  |
