# Supplementary material for: High titers of thyroid peroxidase antibodies as a potential risk factor for osteoporosis: A cross-sectional NHANES study and bidirectional Mendelian randomization analysis
Source: Medicine (Baltimore). 2026 Jul 24;105(30):e49917. doi: 10.1097/MD.0000000000049917 (PMC13406175; doi:10.1097/MD.0000000000049917)
Supplement: Supplementary file 2 [file medi-105-e49917-s002.docx]

**Supplementary Table 2. Association between TPOAB and TGAB with wrist and hip fracture**

| **Antibody Status** | | | | | | **Model 1: OR (95%CI)** | **P** | **Model 2: OR (95%CI)** | **P** | **Model 3: OR (95%CI)** | **P** |
| --- | --- | --- | --- | --- | --- | --- | --- | --- | --- | --- | --- |
| Wrist | | | TGAB (+) | | | 1.28  (0.61, 2.69) | 0.51 | 1.32  (0.63, 2.75) | 0.44 | 1.34  (0.62, 2.89) | 0.42 |
|  |  |  | TPOAB (+) | | | 1.21  (0.83, 1.76) | 0.31 | 1.39  (0.93, 2.06) | 0.10 | 1.39  (0.94, 2.06) | 0.09 |
|  |  |  | TGAB&TPOAB (+) | | | 1.78  (0.75, 1.76) | 0.31 | 1.98  (0.86, 4.56) | 0.10 | 2.04  (0.84, 4.97) | 0.10 |
| Hip | | | | TGAB (+) | | 0.24  (0.05, 1.30) | 0.10 | 0.14  (0.02, 4.51) | 0.14 | 0.11  (0.02, 1.80) | 0.13 |
|  |  |  |  | TPOAB (+) | | 0.65  (0.20, 2.09) | 0.46 | 0.39  (0.11, 1.44) | 0.15 | 0.35  (0.09, 1.42) | 0.13 |
|  |  |  |  | TGAB&TPOAB (+) | | 0.40  (0.08, 2.09) | 0.27 | 0.26  (0.04, 1.60) | 0.14 | 0.20  (0.02, 1.61) | 0.12 |
|  | | | | | **Titer level** | **Model 1: OR (95%CI)** | **P** | **Model 2: OR (95%CI)** | **P** | **Model 3: OR (95%CI)** | **P** |
| Wrist | | TGAB | Negative | | | Reference |  | Reference |  | Reference |  |
|  |  |  | Low-titer Group | | | 1.51  (0.71, 3.22) | 0.28 | 1.57  (0.74, 3.33) | 0.67 | 1.58  (0.71, 3.53) | 0.23 |
|  |  |  | High-titer Group | | | 1.62  (0.52, 4.37) | 0.31 | 2.13  (0.81, 5.01) | 0.45 | 3.14  (0.86, 5.12) | 0.47 |
|  |  |  | P for trend | | |  | 0.61 |  | 0.53 |  | 0.49 |
| Wrist | TPOAB | | Negative | | | Reference |  | Reference |  | Reference |  |
|  |  |  | Low-titer Group | | | 1.30  (0.89, 1.89) | 0.17 | 1.50  (0.92, 2.24) | 0.09 | 1.50  (0.97, 2.24) | 0.09 |
|  |  |  | High-titer Group | | | 0.07  (0.01, 1.57) | 0.23 | 0.09  (0.01, 1.09) | 0.08 | 0.09  (0.01, 1.37) | 0.08 |
|  |  |  | P for trend | | |  | 0.54 |  | 0.2 |  | 0.19 |
| Hip | TGAB | | Negative | | | Reference |  | Reference |  | Reference |  |
|  |  | | Low-titer Group | | | 0.28  (0.05, 1.51) | 0.13 | 0.17  (0.03, 1.04) | 0.08 | 0.13  (0.02, 1.02) | 0.06 |
|  |  | | High-titer Group | | | 0.45  (0.07, 2.11) | 0.32 | 0.34  (0.10, 3.65) | 0.32 | 0.56  (0.01, 4.53) | 0.21 |
|  |  | | P for trend | | |  | 0.1 |  | 0.23 |  | 0.15 |
| Hip | TPOAB | | Negative | | | Reference |  | Reference |  | Reference |  |
|  |  | | Low-titer Group | | | 0.70  (0.21, 2.26) | 0.53 | 0.40  (0.11, 1.50) | 0.16 | 0.36  (0.09, 1.49) | 0.14 |
|  |  | | High-titer Group | | | 0.89  (0.01, 3.21) | 0.62 | 0.52  (0.23, 2.13) | 0.21 | 0.52  (0.09, 1.42) | 0.15 |
|  |  | | P for trend | | |  | 0.4 |  | 0.14 |  | 0.12 |
