# Supplementary material for: High titers of thyroid peroxidase antibodies as a potential risk factor for osteoporosis: A cross-sectional NHANES study and bidirectional Mendelian randomization analysis
Source: Medicine (Baltimore). 2026 Jul 24;105(30):e49917. doi: 10.1097/MD.0000000000049917 (PMC13406175; doi:10.1097/MD.0000000000049917)
Supplement: Supplementary file 3 [file medi-105-e49917-s003.docx]

**Supplementary Table 3. MR estimates from each method of assessing the causal effect of OP on HT**

| **Method** | **β** | **SE** | **OR (95%CI)** | **P** | **Heterogeneity test** | **MR-Egger intercept** | |
| --- | --- | --- | --- | --- | --- | --- | --- |
| IVW | 0.224 | 0.120 | 1.251  (0.989, 1.583) | 0.062 | Q=2.313, P=0.315 |  |  |
| MR Egger | 0.643 | 0.307 | 1.902  (1.042, 3.473) | 0.284 | Q=0.169, P=0.681 | P=0.381 |  |
| Weighted median | 0.294 | 0.146 | 1.342  (0.989, 1.583) | 0.045 |  |  |  |
| Simple mode | 0.345 | 0.185 | 1.411  (0.983, 2.027) | 0.203 |  |  |  |
| Weighted mode | 0.349 | 0.195 | 1.418  (0.968, 2.077) | 0.215 |  |  |  |
| P value for MR-PRESSO global test = 0.305 | | | | | | |  |
