# Supplementary figures and images for: High titers of thyroid peroxidase antibodies as a potential risk factor for osteoporosis: A cross-sectional NHANES study and bidirectional Mendelian randomization analysis
Source: Medicine (Baltimore). 2026 Jul 24;105(30):e49917. doi: 10.1097/MD.0000000000049917 (PMC13406175; doi:10.1097/MD.0000000000049917)

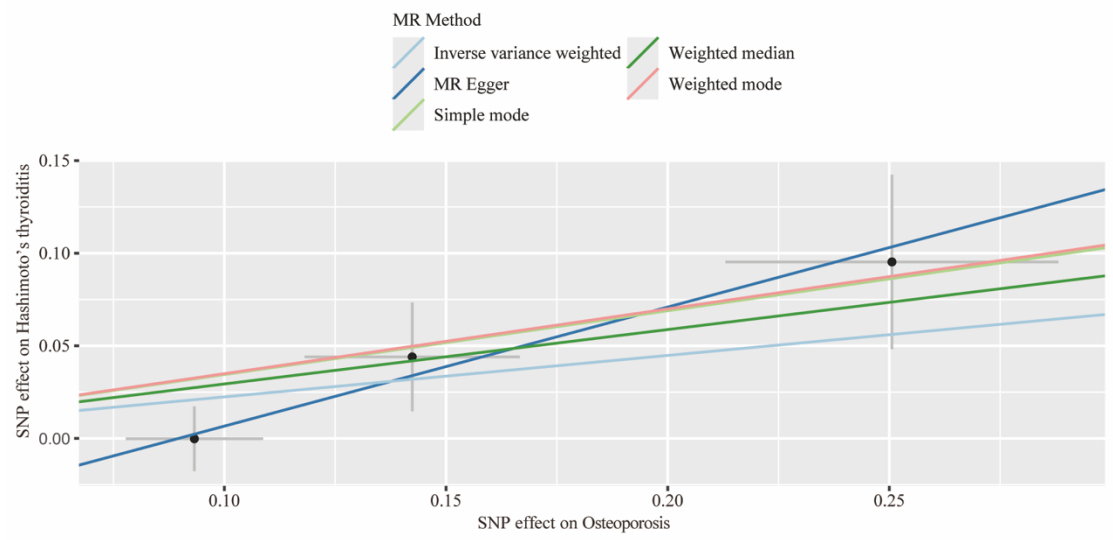

Supplement: Supplementary file 4 [file medi-105-e49917-s004.pdf]

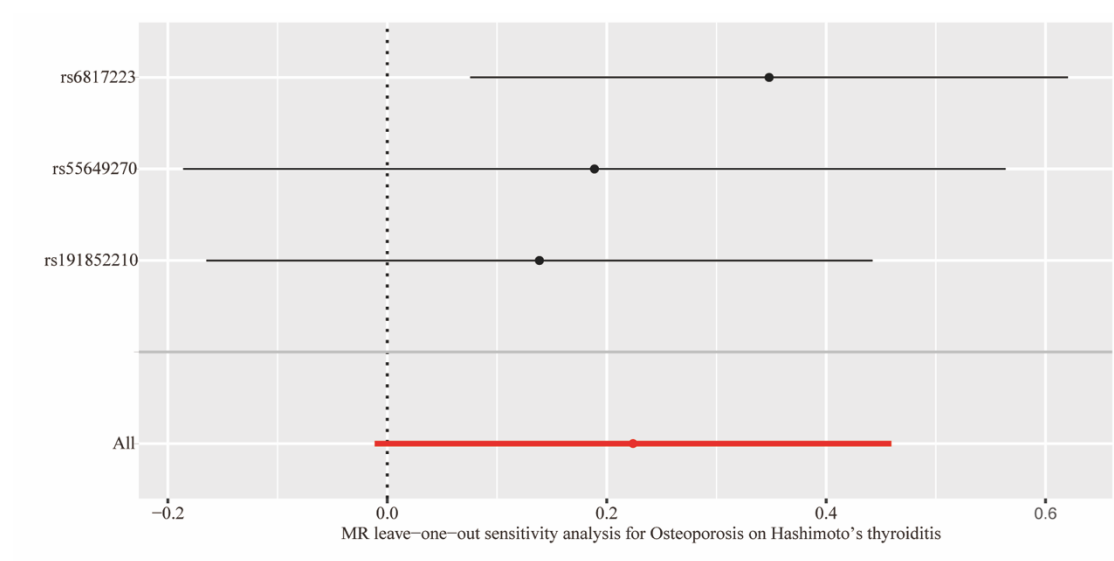

Supplement: Supplementary file 5 [file medi-105-e49917-s005.pdf]
